# Bactericidal Properties of Proline-Rich *Aedes aegypti* Trypsin Modulating Oostatic Factor (*Aea*TMOF)

**DOI:** 10.3390/life13010019

**Published:** 2022-12-21

**Authors:** Dov Borovsky, Pierre Rougé, Robert G. Shatters

**Affiliations:** 1 Department of Biochemistry and Molecular Genetics, University of Colorado Anschutz Medical Campus, Aurora, CO 80045, USA; 2 Faculte des Sciences Pharmaceutiques, 3106 Toulouse, France; 3 USDA-ARS, Horticultural Research Laboratory, Ft. Pierce, FL 34945, USA

**Keywords:** Gram-positive and -negative bacteria, 3D modeling, inhibition of bacterial growth with TMOF and oncocin112 (1–13), bacterial transporters SbmA and MdtM

## Abstract

The antimicrobial properties of proline-rich *Aedes aegypti* decapeptide TMOF (*Aea*TMOF) and oncocin112 (1–13) were compared. Incubations with multidrug-resistant *Escherichia coli* cells showed that *Aea*TMOF (5 mM) was able to completely inhibit bacterial cell growth, whereas oncocin112 (1–13) (20 mM) partially inhibited bacterial growth as compared with bacterial cells that were not multidrug-resistant cells. *Aea*TMOF (5 mM) was very effective against *Acinetobacter baumannii* and *Pseudomonas aeruginosa*, completely inhibiting cell growth during 15 h incubations. *Aea*TMOF (5 mM) completely inhibited the Gram-positive bacteria *Staphylococcus aureus* and *Bacillus thurengiensis* sups. *Israelensis* cell growth, whereas oncocin112 (1–13) (10 and 20 mM) failed to affect bacterial cell growth. *E. coli* cells that lack the SbmA transporter were inhibited by *Aea*TMOF (5 mM) and not by oncocin112 (1–13) (10 to 20 mM), indicating that *Aea*TMOF can use other bacterial transporters than SbmA that is mainly used by proline-rich antimicrobial peptides. Incubation of *E. coli* cells with NaAzide showed that *Aea*TMOF does not use ABC-like transporters that use ATP hydrolysis to import molecules into bacterial cells. Three-dimensional modeling and docking of AeaTMOF to SbmA and MdtM transporters showed that *Aea*TMOF can bind these proteins, and the binding location of AeaTMOF inside these protein transporters allows *Aea*TMOF to be transported into the bacterial cytosol. These results show that *Aea*TMOF can be used as a future antibacterial agent against both multidrug-resistant Gram-positive and -negative bacteria.

## 1. Introduction

Antibiotics have enhanced the treatment against bacterial infections preventing bacterial epidemics. This accomplishment, however, is now challenged by increase of bacterial resistance that has allowed bacteria to overcome almost all currently used antibiotics [1]. Although a partial success has been reported against Gram-positive pathogens that are methicillin-resistant *Staphylococcus aureus* (MRSA) [2], growing concerns exist for Gram-negative pathogens that are resistant, such as *Escherichia coli*, *Klebsiella pneumoniae*, *Enterobacter cloacae*, *Acinetobacter baumannii*, and *Pseudomonas aeruginosa* [1]. Therefore, novel compounds are needed for clinical treatment. Antimicrobial peptides are a promising alternatives for pharmaceutical use. Proline-rich antimicrobial peptides (PrAMPs) that do not employ lytic mechanisms but bind to specific bacterial targets, avoiding human targets, are particularly promising [3]. PrAMPs, that are expressed in mammals and insects, either in their native or as chemically optimized forms have been investigated [4]. Although PrAMPs show low sequence homology, their sequence contains proline (about 30%) and many have Pro–Arg–Pro motifs and use similar mechanisms to kill bacteria [3]. They enter the outer membrane freely before being transported by a polypeptide transporter (SbmA) into the bacterial cytosol [5]. The World Health Organization (WHO) has identified antimicrobial resistance as a major threat to human health [6]. Because bacterial cells are efficient in synthesizing and sharing genes involved in antibiotic resistance, this adverse property causes failure in the treatment of bacterial infections [7,8]. Global antibiotic resistance, along with the misuses of these antibiotic drugs, makes drug design an urgent and paramount research-field [9].

Antimicrobial peptides (AMPs) and PrAMPs are alternatives to current conventional antibacterial treatments [10]; they are synthesized by the host’s innate immune system during an infection [11] by many tissues and cell types by plants, invertebrates, vertebrates, fungi, and bacteria [12]. The majority of AMPs and PrAMPS are small peptides (<10 kDa) that are cationic and amphipathic molecules of 6 to 50 amino acids [13]. Moreover, AMPs and PrAMPs have diverse antimicrobial activities [14]. Members of PrAMPs are found in the hemolymph of several insect species and crustaceans and are also found in the neutrophils of many mammals [15]. They exhibit antimicrobial activities against many Gram-negative bacteria and are potential future antimicrobial agents [16]. Insect PrAMPs include apidaecins (GNNRPVYIPQPRPPHPRL) that are produced by bees (*Apis melifera*) and wasps (*Apis Vespidae*), pyrrhocoricin (VDKGSYLPRPTPPRPIYNRN) produced by firebugs (*Pyrrhocoris apterus*), drosocins (GKPRPYSPRPTSHPRPIRV) produced by fruit flies (*Drosophila*), metalnikowins (VDKPDYRPRRPRPPNM) produced by the green shield bug (*Palomena prasina*) and the milkweed bug (*Oncopeltus fasciatus*) oncocins (VDKPPYLPRPRPPRRIYNR-NH_2_) [15,17,18]. The insect-derived PrAMPs apidaecin and oncocin, and the mammalian Bac7, enter the bacterial cytosol using the SbmA transporter found in many Gram-negative bacteria [19,20]. Apidaecin, oncocin, and Bac7 bind to the ribosome and inhibit protein translation [21,22]. The crystal structure of the oncocin derivative onc112 (VDKPPYLPRPRPPR(_D_-R)IYN(_D_-R)-NH_2_) in complex with the bacterial 70S ribosome showed that onc112 binds to the ribosomal exit tunnel blocking the binding of the aminoacyl-tRNA to the A-site and stopping protein translation [23,24]. *Aea*TMOF is a proline-rich amphipathic decapeptide (YDPAPPPPPP) analogous to the PrAMP that was reported in insects and mammals that use the SbmA transporter to enter the bacterial cell, bind to the bacterial ribosome, and inhibit protein translation [21,22,23,24]. Because *Aea*TMOF is a proline-rich peptide and like onc112 also affects the translation of the mRNA by the ribosomes [25], we tested the possibility that *Aea*TMOF can be used as a novel antimicrobial agent against Gram-negative and -positive bacterial cells. We report here for the first-time in vivo studies and 3D modeling showing that *Aea*TMOF inhibits both Gram-positive and Gram-negative bacterial cell growth using SbmA as well as MdtM transporters [19,20].

## 2. Materials and Methods

### 2.1. Bacterial Strains, and Chemicals

*E. coli* CGSC strain7636: F^−^ Δ(*araD-araB*)*567*, Δ*lac*Z4787(::rrnB-3), *l*^−^, *rph-1*, Δ(*rhaD-rhaB)568*, *hsdR514*, and *E. coli* CGSC strain 8547:

F^−^ Δ*(araD-araB)567*, Δ*lacZ4787*(::rrnB-3), Δ*sbmA742::kan, l*^−^, *rph-1*, Δ*(rhaD-rhaB)568*, *hsdR514* were obtained from CGSC Yale University (http://cgsc.biology.yale.edu/StrainRpt.php (accessed on 11 November 2022)). *E. coli* Strain GC10 *amp* was transformed with plasmid BM4283 *tet*, *kan* into *E. coli amp*, *tet*, *kan*. *Staphylococcus aureus* HG001, AH2183, *Acinetobacter baumannii* AH 8119 ATCC 196061 and *Pseudomonas aeruginosa* PA01, AW5132 were provided by Professor Alexander Horsewill at the University of Colorado Anschutz Medical School Department of Microbiology and Immunology. *Bacillus thuringiensis* subsp. *Israelensis* was provided by Professor Arieh Zaritsky at the Ben Gurion University of the Negev Department of Life Sciences, Israel. The bacterial strains were grown in Luria–Bertani (LB) at 37 °C under aerobic conditions with the addition, when required, of the following antibiotics at concentrations of 100 μg/mL for ampicillin, 50 μg/mL for kanamycin and tetracycline. Sodium azide (NaAzide) was purchased from Sigma-Aldrich (St. Louis, MO, USA). Synthetic *Aea*TMOF (H-YDPAPPPPPP-OH) and a short oncocin112(1–13) (H-VDKPPYLPRPRPP-OH) were purified by HPLC [26]. After HPLC purifications, the TFA ions were exchanged with phosphate ions.

### 2.2. Bacterial Growth in the Presence of AeaTMOF and Oncocin112 (1–13)

*E. coli* cells CGSC strain7636 (*SbmA^+^*), *E. coli* cells CGSC strain 8547 (*SbmA*^−^), *Staphylococcus aureus* cells HG001, AH2183, *A. baumannii* cells AH 8119 ATCC 196061, *P. aeruginosa* cells PA01, AW5132, and *B. thuringiensis* subsp. *Israelensis* (10^6^ cpu/mL) were each incubated in 96-well plate containing 100 μL/well of LB medium and different concentrations of *Aea*TMOF and oncocin112 (1–13) in different wells. The plates were incubated at 37 °C for 15 h and bacterial growth were followed at 1 h intervals using absorbance at 630 nm and a Biotech ElX808 microplate reader. Control wells contained bacterial cells without *Aea*TMOF or oncocin112 (1–13). To find out if *Aea*TMOF used ABC transporter in *E. coli* cells that lack the SbmA transporter, *E. coli* (*SbmA*^−^) cells were incubated for 18 h at 37° in LB medium as above in the presence of NaAzide (25 and 250 μM) and AeaTMOF (5 mM) and the absorbance at 630 nm was determined. At the end of the incubation period, aliquots (0.5 μL) were removed from each well and spread on LB agar plates that were incubated overnight at 37 °C and viable colonies were counted. Incubations were performed in triplicates and the experiments were repeated twice. Results are expressed as averages of three determinations + SEM.

### 2.3. Molecular Modeling and Docking of AeaTMOF

#### 2.3.1. SbmA Transporter

Homology modeling of the ABC transporter corresponding to the *sbmA* gene of *Escherichia coli* K-12 (accession number WP_001304845.1) was conducted with the YASARA Structure program [27]. The SbmA model was built from the X-ray coordinates of the *Staphylococcus aureus* ABC transporter (PDB code 2HYD) [28] as a template. The geometric quality of the model was assessed using PROCHECK [29]. The residues of SbmA were correctly assigned to the allowed regions in the Ramachandran plot except for H229 and A290, which occupy a non-allowed region of the plot. Using ANOLEA [30] to evaluate the model, only 10 residues of SbmA out of 402 exhibited an energy over the threshold value. These residues are located in the loop region connecting α-helices and β-sheets. The calculated QMEAN score for the SbmA model is −3.92 [31,32]. Atomic coordinates of the *E. coli* ABC transporter MalFGK_2_ (PDB code 4KHZ) [33], and *E. coli* ABC transporter McjD (PDB code 5OFP) [34], were obtained from the PDB [35].

The hydrophilic and hydrophobic regions distributed on the surface of SbmA were identified using Chimera [36]. The coulombic charges on the surface of SbmA were calculated using the classic values for the inner and outer dielectric constants obtained from to the proteins and the solvent (4.0 and 80.0, respectively).

The decapeptide *Aea*TMOF (YDPAPPPPPP) was built as a left-handed α-helix [37] using Chimera [36], and the structure was minimized by 1000 steps of steepest descent and 100 steps of conjugated gradient. Docking of *Aea*TMOF to SbmA was performed by using YASARA [27]. Additional docking experiments were also submitted to the GRAMM_X [38,39] web server. Molecular cartoons were drawn and rendered using YASARA Structure, Chimera, and ChimeraX programs [36].

#### 2.3.2. MdtM Transporter

The *E. coli* membrane transporter MdtM was modeled using YASARA [27], and the following templates: *E. coli* multidrug transporter MtdA in complex with deoxycholate (PDB code 4ZP0) [40], the *E. coli* multidrug transporter Emrd (PDB code 2GFP) [41], the YajR transporter from *E. coli* (PDB code 3WDO) [42], and the *E. coli* POT transporter (PDB code 6EI3) [43]. A hybrid model was built and used as the final model for the *E. coli* MdtM transporter. PROCHECK [28] was used to assess the geometric quality of the model. All the residues of the modeled MdtM were correctly assigned in the allowed regions in the Ramachandran plot except for two residues, H8 and A195, which occur in a non-allowed region of the plot. The calculated QMEAN score for the MdtM model has an acceptable value of 0.71 [31,32]. Atomic coordinates of the *E. coli* transporters were retrieved from the PDB [35]. The decapeptide *Aea*TMOF (YDPAPPPPPP) was built as a left-handed α-helix as was described above (Section 2.3.1). Docking of *Aea*TMOF to MdtM and POT (proton coupled) transporters was performed with YASARA. Additional docking experiments were submitted to the GRAMM_X [38,39] web server. Molecular cartoons were drawn and rendered as was described above for the SbmA transporter (Section 2.3.1) [44].

### 2.4. Statistical Analysis

Statistical analyses were conducted using GraphPad Prism and two-tailed unpaired student’s *t*-test. Results were considered significant when *p <* 0.05. Each experimental point is an average of 3 determinations ± SEM.

## 3. Results

### 3.1. Gram Negative Bacteria

#### 3.1.1. Effect on *E. coli* (Amp^R^, Tet^R^, Kan^R^)

**Figure 1:**
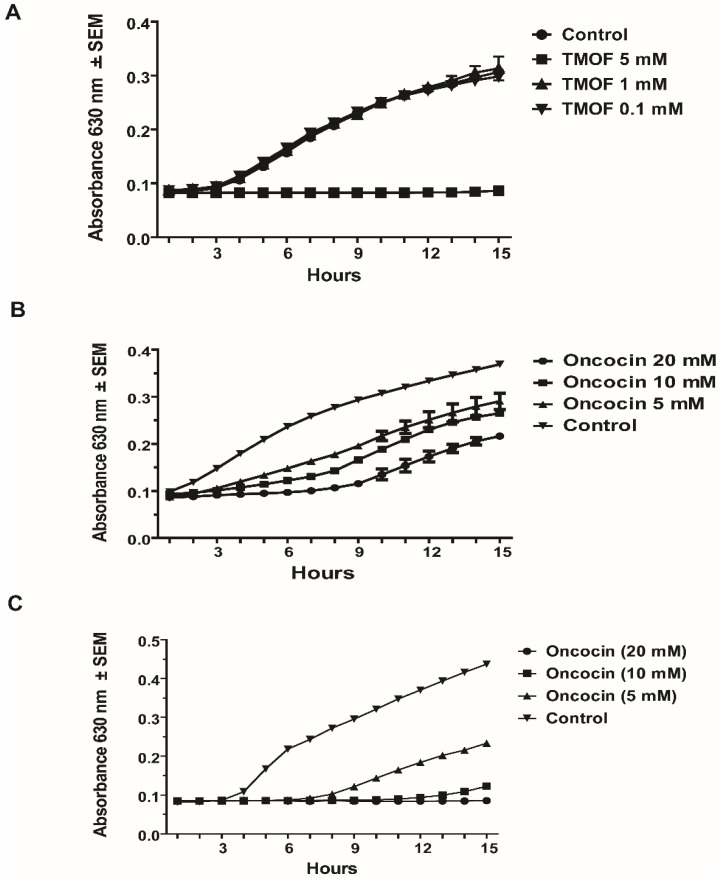
Multidrug-resistant *E. coli* Amp^R^, Kan^R^, Tet^R^ (10^6^ cpu/mL) cell growth in the presence of different concentrations of (**A**). *Aea*TMOF, (**B**). oncocin112 (1–13), (**C**). growth of *E. coli* (not drug resistant) (10^6^ cpu/mL) in the presence of oncocin112 (1–13).

*E. coli* cells (10^6^ cpu/mL) that exhibit multi drug resistance were grown in 100 μL of LB medium containing ampicillin (100 μg/mL), kanamycin, and tetracycline (50 μg/mL/each) in a 96 well-plate at 37 °C for 15 h. Incubation of the bacterial cells with different concentrations of TMOF (0.1 to 5 mM) showed that *Aea*TMOF (5 mM) completely stopped bacterial growth and caused 99.9% cell death; 1–2 cpu were detected at 15 h as compared with 10^6^ cpu/mL that were inoculated into each well. Lower concentrations of TMOF (0.1 and 1 mM, respectively) were not effective (Figure 1A). On the other hand, oncocin112 (1–13) concentrations of 5, 10, and 20 mM were not effective, although 20 mM oncocin112 (1–13) stopped bacterial growth for 8 h; however, after 15 h the cells stated to rapidly grow. Cell growth, however, was 1.9-, 1.4-, and 1.2-fold lower for concentrations of 20 mM, 10 mM, and 5 mM oncocin112 (1–13), respectively, when compared with cells that were grown without oncocin112 (1–13) (Figure 1B). Oncocin112 (1–13) was much more effective when incubated with *E. coli* cells that were not multi-drug resistant. It completely stopped bacterial growth at 20 mM, causing 90% inhibition at 10 mM and 50% inhibition at 5 mM after 15 h incubations as compared with control cells that were incubated without onconcin112 (1–13) (Figure 1C).

#### 3.1.2. Effect on *A. baumannii*

**Figure 2:**
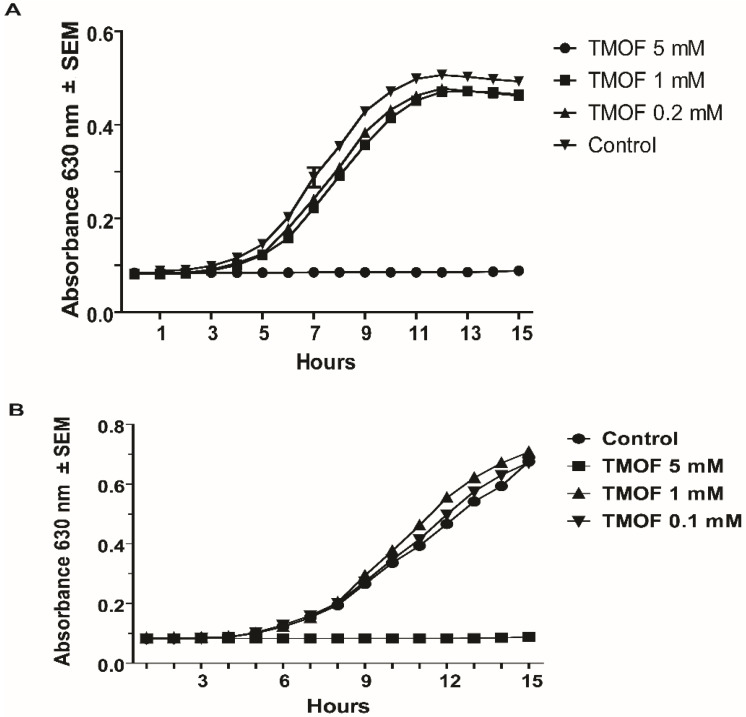
Effect of different concentrations of *Aea*TMOF on (**A**) *A. baumannii* (10^6^ cpu/mL) cell growth, and (**B**) *P. aeruginosa* (10^6^ cpu/mL) cell growth.

To find out if *Aea*TMOF inhibits the growth of *A. baumanni*, a bacterial pathogen that is primarily associated with hospital-acquired infection exhibiting multidrug resistance. *A. baumanni* cells (10^6^ cpu/mL) were incubated with different concentrations of *Aea*TMOF (0.2, 1 and 5 mM). Control cells were incubated without *Aea*TMOF. Bacterial cells that were incubated with *Aea*TMOF did not grow and 99.9% of the cells were dead at 15 h. *Aea*TMOF concentrations (0.2 mM and 1 mM) were not effective, and bacterial cells that were incubated with these *Aea*TMOF concentrations grew at a similar rate as control cells that were not treated with *Aea*TMOF (Figure 2A).

#### 3.1.3. Effect on *P. aeruginosa*

To find out if *Aea*TMOF inhibits the growth of *P. aeruginosa*, a multidrug-resistant pathogen associated with serious hospital-acquired infections. *P. aeruginosa* cells (10^6^ cpu/mL) were incubated with *Aea*TMOF (0.1 mM, 1 mM, and 5 mM) for 15 h. *Aea*TMOF (5 mM) completely stopped bacterial growth, and at the end of the incubation period (15 h), 99.9% of the bacterial cells were dead. Lower concentrations, however, of 0.1 mM and 1 mM did not inhibit bacterial growth and cells grew at a similar rate to control cells that were not incubated with *Aea*TMOF (Figure 2B).

### 3.2. Gram Positive Bacteria

#### 3.2.1. Effect on *S. aureus*

**Figure 3:**
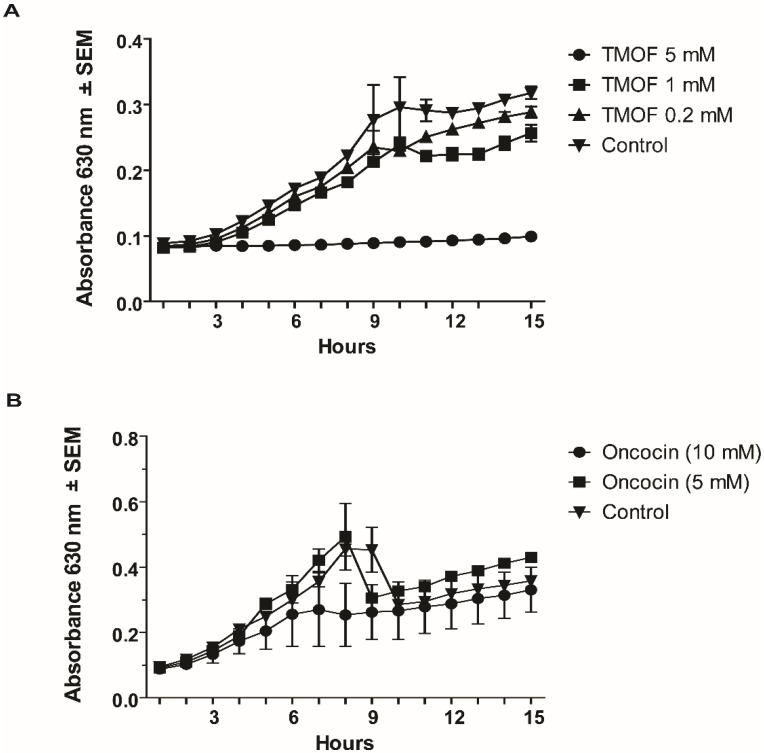
Effect on Gram-positive *S. aureus* (10^6^ cpu/mL) cell growth by different concentrations of (**A**) *Aea*TMOF and (**B**) oncocin112 (1–13).

To find out if *Aea*TMOF affects *S. aureus*, a bacterium that causes wide variety of clinical disease including the drug resistant MRSA (Methicillin Resistant *S. aureus*) strains that invade internal tissues or the blood stream, *S. aureus* (10^6^ cpu/mL) were grown in the presence of *Aea*TMOF (0.2, 1, and 5 mM) and in the absence of the peptide for 15 h. *Aea*TMOF (5 mM) inhibited bacterial growth, killing 99.9% of the cells. On the other hand, lower concentration of *Aea*TMOF (0.2 and 1 mM) did not inhibit bacterial growth when compared with control cells that were incubated without *Aea*TMOF (Figure 3A). Incubation of oncocin112 (1–13) (5 and 10 mM) with *S. aureus* cells (10^6^ cpu/mL) did not inhibit bacterial growth as compared with controls that were incubated without oncocin112 (1–13). However, between 6 to 9 h there was a slight reduction in cell growth. At 15 h, however, a non-significant difference in the growth rate was observed when compared with control cells that were incubated in the absence of oncocin112 (1–13) (Figure 3B).

#### 3.2.2. Effect on *B. thuringiensis* subsp. *Israelensis*

**Figure 4:**
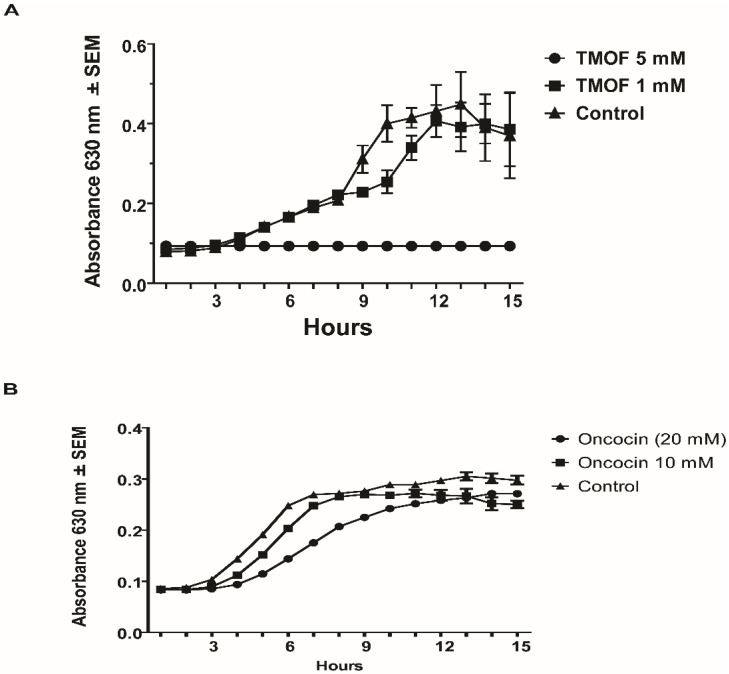
Effect on Gram-positive *B. thuringiensis* subsp. *Israelensis* (10^6^ cpu/mL) cell growth by different concentrations of (**A**) *Aea*TMOF and (**B**) oncocin112 (1–13). *Aea*TMOF (0.2 mM) is not shown in A, as it is similar to the control.

To find out whether *Aea*TMOF can inhibit the growth of *B. thurengiensis* subsp. *Israelenis*, a bacterium that produces Cry and Cyt toxins for the control of mosquito larvae, different concentrations of *Aea*TMOF (1 and 5 mM) were incubated with the bacterial cells (10^6^ cpu/mL). A control was incubated without *Aea*TMOF for 15 h. Bacterial cells that were incubated in the presence of *Aea*TMOF (5 mM) did not grow, and 99.9% of the initial bacterial cells were dead. On the other hand, cells that were incubated in the presence of *Aea*TMOF (1 mM) or without *Aea*TMOF grew at a similar growth-rate, and at 15 h the number of bacterial cells grown with *Aea*TMOF and control cells were not significantly different (Figure 4A). To find out if oncocin112 (1–13) also affects the bacterial cell growth, *B. thuringiensis* subsp. *israelensis* (10^6^ cpu/mL) were incubated with different concentrations of oncocin112 (1–13) (10 and 20 mM) and bacterial growth was monitored at 1 h intervals as above. Bacterial cells that were incubated without oncocin112 (1–13) served as a control group. Although there was a slight growth inhibition initially when onconcin112 (1–13) (20 mM) was incubated with the bacterial cells, at 15 h all the cells grew similarly as compared with the control group that was incubated without oncocin112 (1–13) (Figure 4B). These results indicate that oncocin112 (1–13) does not affect this bacterium’s growth, as was shown for *S. aureus* (see above Section 3.2.1).

### 3.3. The Role of the E. coli SbmA Transporter

**Figure 5:**
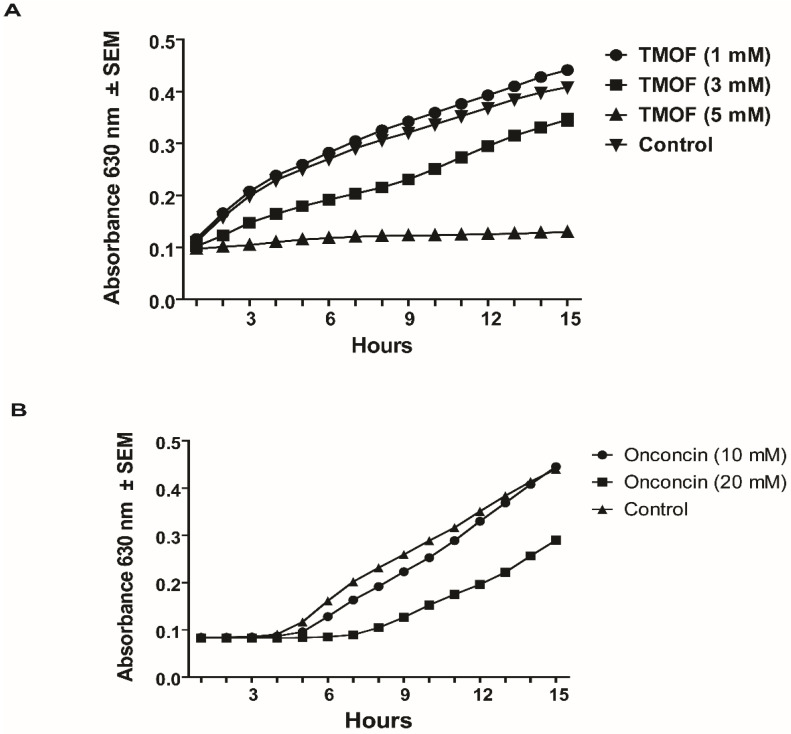
Effect on *E. coli sbmA*^−^ (10^6^ cpu/mL) cell growth by different concentrations of (**A**) *Aea*TMOF and (**B**) oncocin112 (1–13). *Aea*TMOF (0.1 mM) is not shown in A, as it is similar to the control.

SbmA is a bacterial inner membrane protein that forms a dimer and imports into the cytoplasm of *E. coli* peptides, proline-rich peptides, nucleic acids, antisense peptides, and several oligomers [45]. To find out if *E. coli sbmA*^−^ cells lacking the transporter gene are affected by *Aea*TMOF and oncocin112 (1–13), *E. coli* CGSC strain 8547 *sbmA*^−^ (10^6^ cpu/mL) was incubated with different concentrations of *Aea*TMOF (1, 3, and 5 mM) or without TMOF (control) for 15 h, and at 1 h intervals cell growth was determined at 630 nm. *Aea*TMOF (5 mM) completely inhibited bacterial growth and partially inhibited cells growth (23%) at *Aea*TMOF (3 mM) as compared with control cells that were not incubated with *Aea*TMOF, indicating that other importers aside from SbmA transport *Aea*TMOF into the bacterium cell (Figure 5A). On the other hand, incubations with higher concentrations of oncocin112 (1–13) (20 mM) were needed to achieve 43% inhibition in bacterial cell growth after 15 h incubation, and no inhibition was noted at a lower concentration of 10 mM (Figure 5B), indicating that oncocin112 (1–13) is mainly imported into bacterial cells by the SbmA transporter.

### 3.4. E. coli sbmA^−^ Cell Growth in the Presence of AeaTMOF and NaAzide

**Figure 6:**
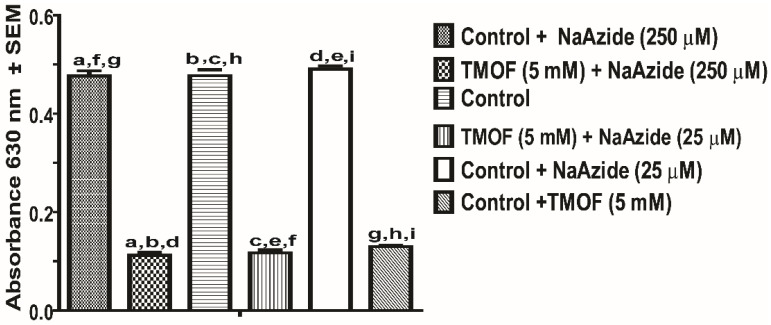
Effect of *Aea*TMOF (5 mM) on *E. coli sbmA*^−^ (10^6^ cpu/mL) cell growth in the presence and absence of NaAzide (25 and 250 mM). Control cells were incubated without *Aea*TMOF and with NaAzide (25 and 250 mM) or without NaAzide and in the presence of *Aea*TMOF (5 mM). Results are expressed as means of three determinations ± SEM. ^a–i^ Significantly different (*p* > 0.0001).

Our results indicate that *Aea*TMOF is imported into *E. coli* cells that lack an *SbmA* importer (Figure 5A). To find out if *Aea*TMOF uses an ABC importer like the *Aea*TMOF receptor/importer in *Ae. aegypti* [46], *E. coli* CGSC strain 8547 *sbmA*^−^ (10^6^ cpu/mL) was incubated for 18 h with *Aea*TMOF in the presence of NaAzide (25 and 250 mM) in order to inhibit ATPase activity that is associated with ABC importers. Controls were incubated without *Aea*TMOF and with NaAzide (50 and 250 mM) or without NaAzide. *E. coli sbmA*^−^ cells that were incubated in the presence of *Aea*TMOF (5 mM) and in the presence of NaAzide (50 and 250 mM) or without NaAzide did not grow and were significantly inhibited as compared with controls (*p* < 0.0001), whereas controls that were incubated without *Aea*TMOF in the presence of NaAzide (50 and 250 mM) or in the absence of NaAzide grew normally and no significant difference was found when the three controls were compared (Figure 6). These results suggest that in the absence of *SbmA*, *Aea*TMOF is using another transporter to enter the bacterial cells.

### 3.5. Docking of AeaTMOF to SbmA and MdtM Transporters

Our results indicate that *Aea*TMOF is imported into bacterial cells using transporters that are not ABC importers. To show that *Aea*TMOF uses SbmA and MdtM importers, both do not use ATP to import proline rich molecules into bacterial cells, we docked *Aea*TMOF to these importers.

#### 3.5.1. SbmA

**Figure 7:**
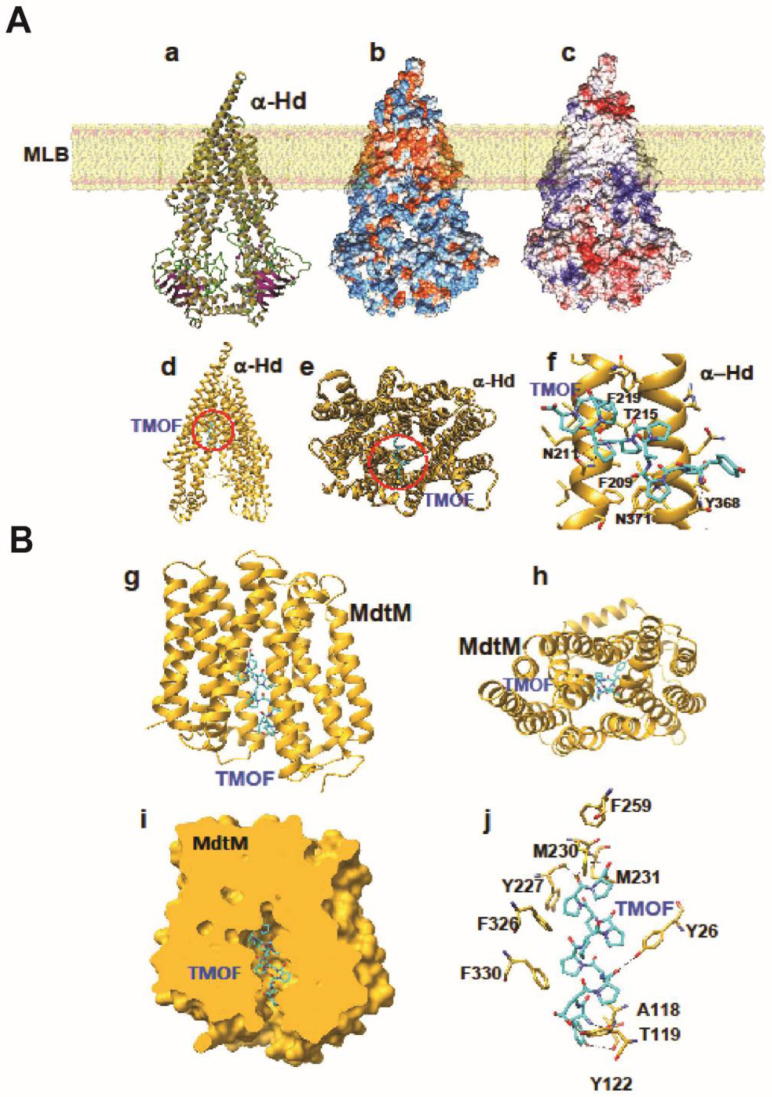
(**A**) Three-dimensional modeling and docking of *Aea*TMOF to *E. coli SbmA*. (**a**) Ribbon diagram of the three-dimensional model built for sbmA inserted in the membrane lipid bilayer (MLB). (**b**) Hydrophilic (colored blue) and hydrophobic (colored orange) patches distributed on the molecular surface of SbmA. The hydrophobic character of α-helices allowing α-Hd to become embedded into the membrane lipid bilayer (MLB). (**c**) Distribution of coulombic charges (electronegative and electropositive charged regions are colored red and blue, respectively; neutral regions are white) on the molecular surface of α-Hd. (**d**) Ribbon diagram showing the upper part of *E. coli* SbmA and the position of *Aea*TMOF (cyan-colored sticks) docked into the binding-site located in the fork of the α-Hd. (**e**) Front view of α-Hd containing the docked position (red dashed circle) of *Aea*TMOF (cyan-colored sticks). (**f**) Position of *Aea*TMOF (colored cyan) in the binding-cavity in the α-helical domain α-Hd of SbmA. Hydrogen bonds anchoring *Aea*TMOF to N211, T215, and N371 of the α-Hd are represented by black dashed lines. Aromatic residues (F209, F219, Y368) participating in the stacking interaction between *Aea*TMOF and α-Hd are shown as broken black lines. (**B**) Three-dimensional modeling and docking of *Aea*TMOF to *E. coli* MdtM. (**g**) Lateral view of the ribbon diagram of MdtM showing TMOF (colored cyan) inserted in the central channel of the receptor. (**h**) Upper view of the ribbon diagram of MdtM showing the insertion of TMOF in the central channel. (**i**) A cut plane of the molecular surface of MdtM with *Aea*TMOF inserted in the central channel. (**j**) Network of hydrogen bonds (broken black lines) anchoring TMOF to residues Y26, A118, T119, Y122, Y227, M230, and M231 of the MdtM receptor. Aromatic residues Y26, Y122, Y227, F259, F326, and F330 participate in stacking interactions with TMOF.

The modeled α-helical domain (α-Hd) of the proline-rich peptide transporter (SbmA) of *E. coli* exhibits a typical V-shaped structure (Figure 7(Aa)), embedded in the membrane lipid bilayer (MLB) via a highly hydrophobic region (Figure 7(Ab)), which extends the α-Hd into an intracellular domain, and linked with a smaller extracellular domain (Figure 7(A(a,b)). Both domains are located on the sides of a hydrophobic α-helical region and are mainly hydrophilic (Figure 7(Ab)), displaying electropositive and electronegative charged patches on their surfaces (Figure 7(Ac)). Docking experiments performed with the *Aea*TMOF decapeptide as a ligand resulted in optimal position of the decapeptide in the substrate-binding cavity located at the fork of the V-shaped α-Hd structure (Figure 7(Ad–f)). The binding-site accommodating the *Aea*TMOF decapeptide exhibits electrostatic, hydrophilic, hydrophobic, and stacking interactions, including a few hydrogen bonds that participate in anchoring *Aea*TMOF to the binding site (Figure 7(Af)). The α-helices and loops at the top of the extracellular α-helical domain are sufficiently open to allow flexibility that allows access to *Aea*TMOF and subsequent anchoring of the molecule to the binding site located in the intracellular part of *E. coli* SbmA, at the fork of α-Hd (Figure 7(Ad–f)).

#### 3.5.2. MdtM

The modeled membrane transporter of *E. coli* MdtM exhibits a canonical organization of membrane transporters and consists of 12 α-helices spanning the lipid bilayer (Figure 7(Bg)). The α-helices delineate a central channel that allows transport of peptides across the bacterial membrane (Figure 7(Bh)). Docking experiments show that *Aea*TMOF readily interacts with the transporter via hydrogen bonds and stacking interactions with aromatic residues surrounding the central channel of MdtM (Figure 7(Bj)). Anchoring of *Aea*TMOF to MdtM involves a network of hydrogen bonds to Y26, A118, T119, Y122, Y227, M230, and M231. Additional stacking interactions between the proline and tyrosine rings of *Aea*TMOF and aromatic residues Y26, Y122, Y227, F259, F326, and F330 of MdtM strengthen the anchoring of TMOF to MdtM (Figure 7(Bj)). The modeling results show that *Aea*TMOF has high affinity for both SbmA and MdtM indicating that they could be used to transport *Aea*TMOF into bacterial cells.

#### 3.5.3. AeaTMOF and Oncocin112 (1–13) Binding

**Figure 8:**
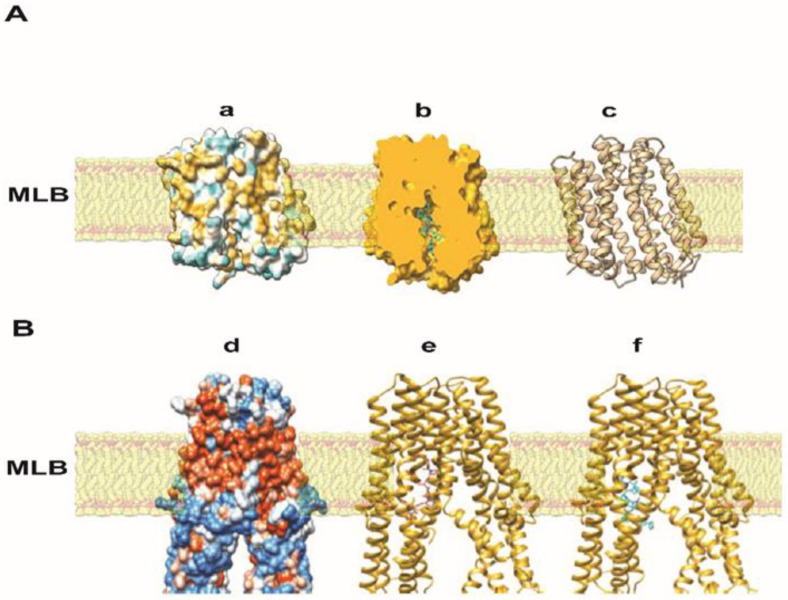
Insertion of MdtM and SbmA into the lipid bilayer of *E. coli* (**A**). (**a**). Upper and lower parts of MdtM are hydrophobic (colored blue) whereas the central region in contact with the membrane lipid bilayer (MLB) is hydrophobic (colored orange); (**b**). cut plane of MdtM, showing the binding of TMOF to the central hydrophobic region of the transporter. (**c**). Ribbon diagram of MdtM showing where the *N*- and *C*-termini are located. The MdtM signal sequence allows the transporter to become inserted into the lipid bilayer, which is linked to the *N*-terminus of the MdtM. (**B**) (**d**). Insertion of SbmA into the MLB of *E. coli* showing the upper part of the molecule through the MLB hydrophilic (colored blue) and hydrophobic (colored orange) patches’ distribution of the molecular surface of sbmA (only upper and middle part are shown); (**e**). docking of oncocin112 (1–13) (colored pink) and (**f**) *Aea*TMOF (colored cyan) to SbmA (colored gold) involves different networks of hydrogen bonds and hydrophobic interactions, which account for the specific binding of both ligands to SbmA because of differences in their structure and positioning towards the SbmA α-helices.

The positions of MdtM and SbmA in the bilayer lipid inner membrane of *E. coli* and the binding of *Aea*TMOF to the transporters and oncocin112 (1–13) to SbmA indicate that both peptides bind the importers inside the membrane at positions that are dictated by charge, hydrophobic, and stacking interactions with the transporters (Figure 8(b,e,f)). Although *Aea*TMOF and oncocin112 (1–13) are bound in the same active site, the binding is different (Figure 8(Be,f)): oncocin112 (1–13) extends towards the top of the bacterial bilayer membrane because of a different 3D conformation, whereas TMOF exhibiting a left-handed helix is more compact binding in the middle of the active pocket at the end of the bacterial bilayer membrane, which is also observed in binding MdtM (Figure 8(Ab,Be,f)).

## 4. Discussion

AeaTMOF (YDPAPPPPPP), oncocin (VDKPPYLPRPRPPRRIYNR-NH_2_), and oncocin112 (VDKPPYLPRPRPPR(_D_-R)IYN(_D_-R)-NH_2_), where R15 and R19 have been substituted with D-amino acids, belong to a family of proline-rich peptides that inhibit the translation of proteins by bacterial ribosomes such as oncocin112 [21,23,24]. To compare the decapeptide (*Aea*TMOF) with oncocin112 (1–19), we used a shorter peptide of 13 amino acids oncocin112 (1–13) that was shown by X-ray crystallography to occupy the bacterial ribosomal exit tunnel at its entrance blocking tRNA movement, mRNA translation, and protein biosynthesis [23,24]. To test the inhibitory effect of *Aea*TMOF and oncocin112 (1–13) on bacterial growth, different concentrations of the peptides were incubated in the presence of antibiotic-resistant (Amp^R^, Kan^R^, and Tet^R^) *E. coli* cells (10^6^ cpu/mL). TMOF at 5 mM completely stopped *E. coli* growth, whereas oncocin112 (1–13) was not effective at 5 mM, and even at 20 mM failed to stop bacterial growth (Figure 1A,B). On the other hand, oncocin112 (1–13) was much more effective in stopping *E. coli* cell growth at 20 mM and 10 mM and less effective when 5 mM concentrations were used, showing that *Aea*TMOF is better in stopping antibiotic resistant *E. coli* growth than oncocin112 (1–13). (Figure 1 A–C).

Because antibiotic resistance is a problem in many hospitals in the USA and beyond, *Aea*TMOF could perhaps be used in the future to control bacterial-resistant strains [1,2]. We also tested the effect of AeaTMOF on two medically important Gram-negative bacteria *A. baumannii* and *P. aeruginosa*. The first causes outbreaks infection in hospital intensive care units (ICUs) and healthcare settings affecting very ill or disabled patients causing pneumonia symptoms, bloodstream infection, wound infection, and urinary tract infection. The second bacterial species causes infections in the blood, lungs (pneumonia), or other parts of the body after surgery. Both bacterial species are known to acquire antibiotic resistance and can become multi-drug resistant [47,48].

*A. baumannii* and *P. aeruginosa* growth was completely inhibited in the presence of *Aea*TMOF (5 mM), and lower concentrations (0.1, 0.2, and 1 mM) were not effective. At 15 h only one or two colonies were found, indicating that *Aea*TMOF is very effective in stopping the growth of these bacterial cells (Figure 2). One explanation why *P. aeruginosa* was not inhibited by oncocin112 (1–13) is because this bacterial species is known to lack the SbmA transporter that is required to import PrAMP including oncocin112 (1–13) [49], whereas *Aea*TMOF apparently can use other transporters and was not affected. *S. aureus*, a Gram-positive bacterium, is a major human pathogen causing a wide range of clinical infections. It plays a major role in bacteremia and infective endocarditis, as well as skin and soft tissue and pleuropulmonary infections that are caused by virulent strains that exhibit β-lactam antibiotics resistance [50]. *Aea*TMOF incubation with these bacterial cells caused 99.9% inhibition of bacterial growth during a 15 h incubation period when *Aea*TMOF (5 mM) was used, lower concentrations (0.2 and 1 mM) were not effective (Figure 3A). Although we did not test the bacterial cells for antibiotic resistance, most of the *S. aureus* strains are currently β-lactam resistant. On the other hand, onconcin112 (1–13) did not affect the bacterial cells’ growth even at high concentration (10 mM) (Figure 3B). Because Gram-positive bacteria generally lack an SbmA transporter, PrAMP peptides such as oncocin112 (1–13) do not enter the bacterial cells and do not affect their growth; on the other hand, *Aea*TMOF, a proline-rich decapeptide, can use other transporters to enter the bacterial cells and affect their growth [51]. Similar results were obtained when Gram-positive *B. thuringiensis* subsp. *israelensis* were grown in the presence of *Aea*TMOF and onconcin112 (1–13). Only *Aea*TMOF (5 mM) was able to completely inhibit bacterial cell growth, whereas onconcin112 (1–13) failed to inhibit bacterial cell growth even at high concentration (20 mM) (Figure 4A,B), indicating that this bacterium lacks a SbmA transporter and thus preventing oncocin112 (1–13) from entering the bacterial cell, whereas *Aea*TMOF uses other transporter(s) to enter the bacterial cells [51]. Indeed, *E. coli* cells, lacking the SbmA transporter, that were incubated with *Aea*TMOF and oncocin112 (1–13) were inhibited only in the presence of *Aea*TMOF (5 mM and 3 mM, respectively) (Figure 5A), whereas concentrations of oncocin112 (1–13) (10 mM and 20 mM) failed to fully inhibit the growth of these bacterial cells (Figure 5B) indicating that oncocin112 (1–13) uses SbmA exclusively to enter the *E. coli* cells, whereas *Aea*TMOF in the absence of SbmA can use other transporters such as MdtM, YjiL, or YgdD to enter the bacterial cells [20]. Our earlier reports [25,46] show that *Aea*TMOF is transported into the midgut epithelial cells of *Ae. Aegypti* by an ABC importer that uses ATP hydrolysis to transport *Aea*TMOF into the mosquito gut epithelial cells before it binds the mosquito ribosome, stopping trypsin biosynthesis [25,46]. To eliminate the possibility that *Aea*TMOF uses a similar transporter to enter *E. coli* cells, we incubated *E. coli* cells lacking the SbmA transporter in the presence of NaAzide (25 and 250 mM) to inhibit ATPase activity, thus inhibiting *Aea*TMOF-like ABC transporters in *E. coli*. Our results show that *Aea*TMOF (5 mM) significantly (*p* < 0.0001) inhibited *E. coli* cell growth in the presence of NaAzide (50 and 250 mM), whereas the growth of bacterial cells (control) that were incubated without *Aea*TMOF and in the presence of NaAzide were not inhibited (Figure 6). These results indicate that *Aea*TMOF in the absence of SbmA uses different transporter(s) [20], although several genes are alleged to be alternative transporters for PrAMP, including the *yjiL-mdtM* gene cluster, which aside from its role as an efflux pump can also be used to import PrAMP into bacterial cells [20]. YjiL is a putative ATPase activator of (R)-hydroxyglutaryl-CoA dehydratase (Gene ID 948837) (www.ncbi.nlm.nih.gov/gene/ (accessed on 10 November 2022)), and its function, although unknown, could possibly facilitate ABC transporters [51]. On the other hand, SbmA and MdtM can each form a channel through the bacterial cytoplasmic membrane to transport proline-rich peptides such as *Aea*TMOF. SbmA 3D modeling shows that it has high similarity to the ABC transporters except for nucleotide binding sites (Figure 7(Aa)) and uses electrochemical gradient and not ATP hydrolysis to import PrAMP into the bacterial cells [5]. Our docking results show that *Aea*TMOF binds SbmA at a specific binding cavity using electrostatic interactions, hydrophilic and hydrophobic interactions, stacking interactions, and a few hydrogen bonds (Figure 7(Ad–f)). The α-helices and the loops at the top of the extracellular part of the α-Hd that extend outside the inner membrane are sufficiently loose and flexible and allow *Aea*TMOF to enter and anchor at the binding site in the fork of the a-HD (Figure 7(Ad,e)), indicating that *Aea*TMOF uses SbmA to enter *E. coli* cells. Similarly, a 3D modeling of MdtM and docking of *Aea*TMOF show that the decapeptide binds the central channel of MdtM using network of hydrogen bonds and stacking interactions between Pro and Tyr rings of *Aea*TMOF and the aromatic residues of MdtM (Figure 7B). *Aea*TMOF exhibited low hemolytic activity when different concentrations of *Aea*TMOF (0–6.5 mM) were tested by Professor Robert Hodges (university of Colorado Anschutz School of Medicine, department of Biochemistry and Molecular Genetics) using females’ (ages 20 to 40 years old) blood samples that were obtained at the University of Colorado Anschutz Medical School Hospital (results not shown). Therefore, in future work we will clone and express SbmA and MdtM and determine the *Aea*TMOF K_D_ for both transporters as was performed for the *Aea*TMOF ABC receptor [45]. To make *Aea*TMOF more amenable for animal and clinical work, it will be necessary in the future to alter the peptide as was achieved for oncocin112 [21], reducing the effective bactericidal concentrations from millimolar to micromolar range. For future clinical work, the peptide should be encapsulated to allow a slow release into the circulation of test animals.

## 5. Conclusions

*Aea*TMOF is a mosquito decapeptide hormone that is synthesized by female mosquito ovaries after the blood meal and secreted into the hemolymph binds a *Aea*TMOF ABC gut receptor and is imported into the gut epithelial cells regulating trypsin biosynthesis by binding the gut ribosomes stopping the translation of the trypsin transcript after the blood meal. Because of this unique property of *Aea*TMOF, we explored the possibility that bacterial transporters SbmA and MdtM can also import *Aea*TMOF into the bacterial cytosol, allowing the peptide to stop protein translation by the bacterial ribosome and cause bacterial death. We first used 3D molecular modeling to show that SbmA and MdtM importers bind and could transport *Aea*TMOF by comparing it with oncocin112 (1–13), a proline-rich peptide that is transported by SbmA into the bacterial cytosol. Oncocin112 (1–13) was shown by X-ray crystallography to bind the bacterial ribosome at the protein exit tunnel blocking mRNA translation. Indeed, incubation of both Gram-negative and -positive bacterial cells in the presence of *Aea*TMOF caused bacterial death. Because *Aea*TMOF exhibits low hemolytic activity, it could be used to control bacterial infections in future.

## Data Availability

All data was provided in the manuscript.

## References

[1] Nolte O. (2014). Antimicrobial Resistance in the 21st Century: A Multifaceted Challenge Protein. Pept. Lett..

[2] Kumar K., Chopra S.J. (2013). New drugs for methicillin-resistant Staphylococcus aureus: An update. Antimicrob. Chemother..

[3] Otvos L. (2002). The short proline-rich antibacterial peptide family. Cell. Mol. Life Sci..

[4] Ostorhazi E., Nemes-Nikodem E., Knappe D., Hoffmann R. (2014). In vivo Efficacy and Pharmacokinetics of Optimized Apidaecin Analogs Protein. Pept. Lett..

[5] Runti G., Lopez Ruiz M.C., Stoilova T., Hussain R., Jennions M., Choudhury H.G., Benincasa M., Gennaro R., Beis K., Scocchi M. (2013). Functional characterization of SbmA, a bacterial inner membrane transporter required for importing the antimicrobial peptide Bac7(1-35). J. Bacteriol..

[6] Shrivastava S., Shrivastava P., Ramasamy J. (2018). World health organization releases global priority list of antibiotic-resistant bacteria to guide research, discovery, and development of new antibiotics. J. Med. Soc..

[7] El-Halfawy O.M., Valvano M.A. (2015). Antimicrobial heteroresistance: An emerging field in need of clarity. Clin. Microbiol. Rev..

[8] Nicoloff H., Hjort K., Levin B.R., Andersson D.I. (2019). The high prevalence of antibiotic heteroresistance in pathogenic bacteria is mainly caused by gene amplification. Nat. Microbiol..

[9] Boucher H.W., Talbot G.H., Bradley J.S., Edwards J.E., Gilbert D., Rice L.B., Scheld M., Spellberg B., Bartlett J. (2009). Bad bugs, no drugs: No ESKAPE! An update from the Infectious Diseases Society of America. Clin. Infect. Dis..

[10] Lai Y., Gallo R.L. (2009). AMPed up immunity: How antimicrobial peptides have multiple roles in immune defense. Trends Immunol..

[11] Hancock R.E., Diamond G. (2000). The role of cationic antimicrobial peptides in innate host defenses. Trends Microbiol..

[12] Brogden K.A. (2005). Antimicrobial peptides: Pore formers or metabolic inhibitors in bacteria?. Nat. Rev. Microbiol..

[13] Bradshaw J.P. (2003). Cationic antimicrobial peptides. BioDrugs.

[14] Izadpanah A., Gallo R.L. (2005). Antimicrobial peptides. J. Am. Acad. Dermatol..

[15] Scocchi M., Tossi A., Gennaro R. (2011). Proline-rich antimicrobial peptides: Converging to a non-lytic mechanism of action. Cell. Mol. Life Sci..

[16] Li W., Tailhades J., O’Brien-Simpson N.M., Separovic F., Otvos L., Hossain M.A., Wade J.D. (2014). Proline-rich antimicrobial peptides: Potential therapeutics against antibiotic-resistant bacteria. Amino Acids.

[17] Ebbensgaard A., Mordhorst H., Overgaard M.T., Nielsen C.G., Aarestrup F.M., Hansen E.B. (2015). Comparative evaluation of the antimicrobial activity of different antimicrobial peptides against a range of pathogenic bacteria. PLoS ONE.

[18] Chernysh S., Cociancich S., Briand J.P., Hetru C., Bulet P. (1996). The inducible antibacterial peptides of the Hemipteran insect Palomena prasina: Identification of a unique family of proline rich peptides and of a novel insect defensin. J. Insect Physiol..

[19] Mattiuzzo M., Bandiera A., Gennaro R., Benincasa M., Pacor S., Antcheva N., Scocchi M. (2007). Role of the Escherichia coli SbmA in the antimicrobial activity of proline-rich peptides. Mol. Microbiol..

[20] Krizsan A., Knappe D., Hoffmann R. (2015). Influence of yjiL and upstream genes on the antibacterial activity of proline-rich antimicrobial peptides overcoming Escherichia coli resistance induced by the missing SbmA transporter system. Antimicrob. Agents Chemother..

[21] Krizsan A., Volke D., Weinert S., Strater N., Knappe D., Hoffmann R. (2014). Insect-derived proline-rich antimicrobial peptides kill bacteria by inhibiting bacterial protein translation at the 70 S ribosome. Angew. Chem. Int. Ed..

[22] Mardirossian M., Grzela R., Giglione C., Meinnel T., Gennaro R., Mergaert P., Scocchi M. (2014). The host antimicrobial peptide Bac7(1–35) binds to bacterial ribosomal proteins and inhibits protein synthesis. Chem. Biol..

[23] Roy R.N., Lomakin I.B., Gagnon M.G., Steitz T.A. (2015). The mechanism of inhibition of protein synthesis by the proline-rich peptide oncocin. Nat. Struct. Mol. Biol..

[24] Seefeldt A.C., Nguyen F., Antunes S., Perebaskine N., Graf M., Arenz S., Inampudi K.K., Douat C., Guichard G., Wilson D.N. (2015). The proline-rich antimicrobial peptide Onc112 inhibits translation by blocking and destabilizing the initiation complex. Nat. Struct. Mol. Biol..

[25] Borovsky D., Rougé P., Shatters R.G. (2022). The Ribosome Is the Ultimate Receptor for Trypsin Modulating Oostatic Factor (TMOF). Biomolecules.

[26] Borovsky D., Carlson D.A., Griffin P.R., Shabanowitz J., Hunt D.F. (1993). Mass Spectrometry and characterization of Aedes aegypti trypsin modulating oostatic factor (TMOF) and its analogs. Insect Biochem. Mol. Biol..

[27] Krieger E., Koraimann G., Vriend G. (2002). Increasing the precision of comparative models with YASARA NOVA–a self-parameterizing force field. Proteins.

[28] Dawson R.J., Locher K.P. (2006). Structure of a bacterial multidrug ABC transporter. Nature.

[29] Laskowski R.A., MacArthur M.W., Moss D.S., Thornton J.M. (1993). PROCHECK: A program to check the stereochemistry of protein structures. J. Appl. Cryst..

[30] Melo F., Feytmans E. (1998). Assessing protein structures with a non-local atomic interaction energy. J. Mol. Biol..

[31] Benkert P., Biasini M., Schwede T. (2011). Toward the estimation of the absolute quality of individual protein structure models. Bioinformatics.

[32] Arnold K., Bordoli L., Kopp J., Schwede T. (2006). The SWISS-MODEL workspace: A web-based environment for protein structure homology modelling. Bioinformatics.

[33] Oldham M.M., Chen S., Chen J. (2013). Structural basis for substrate specificity in the *Escherichia coli* maltose transport system. Proc. Natl. Acad. Sci. USA.

[34] Bountra K., Hagelueken G., Choudhury H.G., Corradi V., El Omari K., Wagner A., Mathavan I., Zirah S., Wahlgren W.Y., Tieleman D.P. (2017). Structural basis for antibacterial peptide self-immunity by the bacterial ABC transporter McjD. EMBO J..

[35] Berman H.M., Westbrook J., Feng Z., Gilliland G., Bhat T.N., Weissig H., Shindyalov I.N., Bourne P.E. (2000). The protein data bank. Nucleic Acids Res..

[36] Pettersen E.F., Goddard T.D., Huang C.C., Couch G.S., Greenblatt D.M., Meng E.C., Ferrin T.E. (2004). UCSF Chimera—A visualization system for exploratory research and analysis. J. Comput. Chem..

[37] Curto E.V., Jarpe M.A., Blalock J.B., Borovsky D., Krishna N.R. (1993). Solution structure of trypsin modulating oostatic factor is a left-handed helix. Biochem. Biophys. Res. Commun..

[38] Tovchigrechko A., Vakser I.A. (2005). Development and testing of an automated approach to protein docking. Proteins.

[39] Tovchigrechko A., Vakser I.A. (2006). GRAMM-X public web server for protein-protein docking. Nucleic Acids Res..

[40] Heng J., Zhao Y., Liu M., Liu Y., Fan J., Wang X., Zhao Y., Zhang X.C. (2015). Substrate-bound structure of the E. coli multidrug resistance transporter MdfA. Cell Res..

[41] Yin Y., He X., Szewczyk P., Nguyen T., Chang G. (2006). Structure of the multidrug transporter EmrD from *Escherichia coli*. Science.

[42] Jiang D., Zhao Y., Wang X., Fan J., Heng J., Liu X., Feng W., Kang X., Huang B., Liu J. (2013). Structure of the YajR transporter suggests a transport mechanism based on the conserved motif A. Proc. Natl. Acad. Sci. USA.

[43] Parker J.L., Li C., Brinth A., Wang Z., Vogeley L., Solcan N., Ledderboge-Vucinic G., Swanson J.M.J., Caffrey M., Voth G.A. (2017). Proton movements and coupling in the POT family of peptide transporters. Proc. Natl. Acad. Sci. USA.

[44] Pettersen E.F., Goddard T.D., Huang C.C., Meng E.C., Couch C.S., Croll T.I., Morris J.H., Ferrin T.E. (2021). UCSF ChimeraX: Structure viszualization for researchers, educators, and developers. Protein Sci..

[45] Corbalan N., Runti G., Adler C., Covaceuszach S., Ford R.C., Lamba D., Beis K., Scocchi M., Vincenta P.A. (2013). Functional and Structural Study of the Dimeric Inner Membrane Protein SbmA. J. Bacteriol..

[46] Borovsky D., Deckers K., Vanhove A.C., Verstraete M., Rouge P., Shatters R.G., Powel C.A. (2021). Cloning and Characterization of Aedes aegypti Trypsin Modulating Oostatic Factor (TMOF) Gut-Receptor. Biomolecules.

[47] Howard A., O’Donoghue M., Audrey Feeney A., Sleator R.D. (2012). Acinetobacter baumannii an emerging opportunistic pathogen. Virulence.

[48] Centers for Disease Control and Prevention (2019). Antibiotic Resistance Threats in the United States.

[49] Runti G., Benincasa M., Giuffrida G., Devescovi G., Venturi V., Gennaro R., Scocchi M. (2017). The mechanism of killing by the prolinerich peptide Bac 7(1–35) against clinical strains of Pseudomonas aeruginosa differs from that against other gram-negative bacteria. Antimicrob. Agents Chemother..

[50] Tong S.Y.C., Davis J.S., Eichenberger E., Holland T.L., Fowler V.G. (2015). Staphylococcus aureus infections: Epidemiology, pathophysiology, clinical manifestations, and management. Clin. Microbiol. Rev..

[51] Frimodt-Moller J., Campion C., Nielsen P.E., Lobner-Olesen A. (2022). Translocation of non-lytic antimicrobial peptides and bacteria penetrating peptides across the inner membrane of the bacterial Envelope. Curr. Genet..

